# Urinary adiponectin concentration is positively associated with micro- and macro-vascular complications

**DOI:** 10.1186/1475-2840-12-137

**Published:** 2013-09-28

**Authors:** Won Seon Jeon, Ji Woo Park, Namseok Lee, Se Eun Park, Eun Jung Rhee, Won Young Lee, Ki Won Oh, Sung Woo Park, Cheol-Young Park, Byung-Soo Youn

**Affiliations:** 1 Division of Endocrinology and Metabolism, Department of Internal Medicine, Kangbuk Samsung Hospital, Sungkyunkwan University School of Medicine, Seoul, Korea; 2 AdipoGen,Inc., Rm 401, Venture Building B, Songdo Technopark, 7-50 Songdo-dong, Yeonsu-gu, Incheon, Republic of Korea

**Keywords:** Adiponectin, Microalbuminuria, Pulse wave velocity

## Abstract

**Background:**

A relationship between plasma adiponectin level and a number of metabolic conditions, including insulin resistance, obesity, and type 2 diabetes, has been reported. This study aimed to assess whether urinary adiponectin concentration is correlated with vascular complications.

**Methods:**

The study comprised 708 subjects who enrolled in the Seoul Metro City Diabetes Prevention Program and were carefully monitored from September 2008 to December 2008. Levels of urinary adiponectin were measured using an enzyme linked immunosorbent assay (ELISA) kit (AdipoGen, Korea). Urinary albumin excretion was assessed by the ratio of urinary albumin to creatinine (A/C ratio). Participants were divided into three groups based on tertiles of urinary adiponectin concentration, and we investigated whether urinary adiponectin levels are associated with microalbuminuria and pulse wave velocity.

**Results:**

Urinary adiponectin concentrations were significantly higher in subjects with microalbuminuria than subjects with normoalbuminuria (*P* < 0.001). Urinary adiponectin concentration was positively correlated with age, fasting plasma glucose level, HbA1C level, triglyceride level, HOMA-IR, systolic/diastolic blood pressure, and urinary A/C ratio (all *P* < 0.05). Subjects in the highest tertile of urinary adiponectin concentration had an increased likelihood of microalbuminuria than those in the lowest tertile (Odds ratio (OR), 6.437; 95% confidence interval (CI), 4.202 to 9.862; *P* < 0.001). After adjusting for age, sex, and estimated creatinine clearance rate (eCcr), the OR remained significant (OR, 5.607; 95% CI, 3.562 to 8.828; *P* < 0.001). Backward multiple linear regression analysis revealed urinary adiponectin concentration to be a significant determinant of mean brachial-ankle pulse wave velocity (baPWV).

**Conclusions:**

An increased urinary adiponectin concentration is significantly associated with microalbuminuria and increased mean baPWV. These results suggest that urinary adiponectin may play an important role as a biomarker for vascular dysfunction.

## Background

Adiponectin, an adipocytokine, is a collagen-like protein expressed mainly in adipose tissue, and accounts for 0.01% of total plasma protein in humans [1]. A relationship between circulating adiponectin level and metabolic conditions such as insulin resistance, obesity, and diabetes mellitus has been demonstrated in previous studies [2-4]. Circulating adiponectin concentration has been shown to be significantly correlated with microalbuminuria and overt nephropathy in type 1 diabetic patients [5,6]. Furthermore, some researchers have reported an association between adiponectin concentration and vascular diseases or arterial stiffness using pulse wave velocity [7-9]. Early detection of vascular damage using markers such as microalbuminuria or arterial stiffness may be important for preventing cardiovascular disease as well as renal complications associated with diabetes mellitus [10]. Several studies have investigated the association between plasma adiponectin concentration and vascular complications. However, the relationship between urinary adiponectin concentration and microalbuminuria or other vascular complication markers has not been fully elucidated [11,12].

In this study, we investigated the relationship between urinary adiponectin concentration and microalbuminuria and arterial stiffness using pulse wave velocity. Furthermore, we examined the association between urinary adiponectin concentration and various metabolic parameters in Korean adults.

## Methods

### Study population

This study included 708 participants who enrolled in the Seoul Metro City Diabetes Prevention Program (SMC-DPP) between September 2008 and December 2008 [13,14]. SMC-DPP is a community-based follow-up program composed of three groups, including healthy subjects and people diagnosed with prediabetes or type 2 diabetes. Participants were recruited from five public health centers and comprised 62 subjects aged 21–86 years with normal glucose tolerance, 250 subjects with prediabetes, and 396 subjects with type 2 diabetes. After exclusion of subjects with previously diagnosed type 2 diabetes or a history of taking any anti-diabetic medication, a 75 g oral glucose tolerance test (OGTT) was performed in the remaining subjects. After the 75 g OGTT was performed, subjects were assigned to one of the groups in accordance with the diagnostic criteria of the American Diabetes Association [15]. Participants with a history of symptomatic heart failure, hepatic dysfunction, severe renal dysfunction (serum creatinine ≥ 2.0 mg/dL), or malignancy were excluded. We also excluded participants with macroalbuminuria (urinary albumin to creatinine ratio > 300 mg/g) in addition to those taking corticosteroids or anti-obesity drugs. We collected information on alcohol intake, smoking status, and use of medications using a structured questionnaire. Brachial-ankle pulse wave velocity (baPWV) was measured in 630 of the 708 study participants. In addition, we investigated serum adiponectin concentrations in 669 subjects. This study protocol was approved by the institutional review board and the ethics committee of Kangbuk Samsung Hospital and was carried out according to the principles of the 1975 Declaration of Helsinki. Written informed consent was obtained from all subjects.

### Anthropometric and laboratory measurements

Systolic and diastolic blood pressures were measured in duplicate and the results averaged. Body mass index (BMI) was calculated by dividing weight in kilograms by the square of height (kg/m^2^). Blood samples were collected to determine plasma glucose, total cholesterol, triglyceride, low-density lipoprotein cholesterol (LDL-cholesterol), and high-density lipoprotein cholesterol (HDL-cholesterol) levels after 12 hours of overnight fasting. Insulin resistance was determined by the homeostasis model assessment index (HOMA-IR). Plasma glucose concentrations were determined using a Beckman glucose analyzer II (Beckman Instruments, Fullerton, CA, USA). HbA1C was determined using high-performance liquid chromatography (Variant II, Bio-Rad Laboratories, Hercules, CA, USA). Lipid profiles were measured by enzymatic colorimetric assay (Siemens, Tarrytown, NY, USA). High-sensitivity C-reactive protein level was measured by nephelometric assay using a BNII nephelometer (Dade Behring, Deerfield, IL, USA).

Urinary and serum adiponectin concentrations were measured using a commercial enzyme linked immunosorbent assay (ELISA) kit (AdipoGen, Korea). Intra-assay coefficients of variation (CVs) for serum ranged between 2.97 and 3.84%, and inter-assay CVs for serum ranged from 2.84 to 5.50%. The intra-assay CVs for urine ranged between 2.12 and 8.31%, while the inter-assay CVs for urine ranged between 3.93 and 9.69%. Urinary adiponectin excretion level was expressed as the ratio of urinary adiponectin concentration to grams of urinary creatinine. Urine albumin excretion was assessed based on the ratio of urinary albumin to creatinine (A/C ratio). To approximate the glomerular filtration rate (GFR), we used the estimated creatinine clearance rate (eCcr) derived by the Cockcroft-Gault formula: eCcr = (140-age)xbody weight(kg) /(serum creatinine(mg/dl)x72)x[0.85 if female].

Pulse wave velocity (PWV) was determined using an automatic waveform analyzer VP2000 (Omron Healthcare Co., Ltd., Kyoto, Japan). Six hundred thirty subjects were examined in the supine position after resting for at least 5 min. Pressure waveforms of the brachial and tibial arteries were recorded by an oscillometric method using occlusion/sensing cuffs applied to both arms and both ankles. We used the mean baPWV (average of right and left brachial-ankle PWVs) value as a marker of arterial stiffness.

### Statistical analysis

Statistical analyses were performed with SPSS version 18.0 (Chicago, IL, USA). For continuous variables, parameters that followed a normal distribution were analyzed by analysis of variance and reported as mean ± standard deviation (SD). Variables with a skewed distribution were log-transformed for statistical analysis. Correlations between adiponectin concentration and other parameters were assessed using Pearson’s correlation analysis. Multiple logistic regression analysis was used to calculate odds ratios for the presence of microalbuminuria (A/C ratio 30-300 mg/g) in subjects in increasing adiponectin tertiles. Backward multiple linear regression analysis was used to evaluate predictive value with mean baPWV as the dependent variable, and age, sex, HOMA-IR, triglyceride level, eCcr, systolic blood pressure, urinary A/C ratio, smoking status, diabetes status, ACEi/ARB use, serum adiponectin, and urinary adiponectin as independent variables. *P* values < 0.05 were considered statistically significant.

## Results

### Baseline characteristics of participants

When study participants were divided into those with normoalbuminuria (A/C ratio <30 mg/g) or microalbuminuria (A/C ratio 30-300 mg/g), mean urinary adiponectin concentration(μg/gCr) was found to be significantly higher in the microalbuminuria group (19.97 ± 27.37) than in the normoalbuminuria group (6.24 ± 9.29)(*P* < 0.001). Mean serum adiponectin concentration(μg/ml) was lower in the microalbuminuria group (7.08 ± 5.75) than in the normoalbuminuria group (8.22 ± 6.23) (log serum adiponectin value: 1.61 ± 0.90 vs. 1.77 ± 0.91, *P* = 0.025).

Participants were also grouped into tertiles based on urinary adiponectin concentration. Subjects in higher urinary adiponectin tertiles were significantly older and had a significantly higher fasting plasma glucose level, HbA1C level, triglyceride level, HOMA-IR, blood pressure, and urinary A/C ratio than subjects in the lower tertiles (all *P* < 0.05). There were significant differences in serum adiponectin concentrations between urinary adiponectin tertiles (*P* < 0.001) (Table 1). When the participants were divided into three groups by their glucose level status (normal glucose status, prediabetes, and diabetes), the mean urinary adiponectin concentrations (μg/gCr) were 2.96 ± 2.03, 9.69 ± 14.96 and 12.52 ± 21.3, respectively (*P* < 0.001). Mean serum adiponectin concentrations (μg/ml) were 7.95 ± 3.25, 8.76 ± 6.19 and 7.25 ± 6.24 (log serum adiponectin value: 1.99 ± 0.39, 1.87 ± 0.86 and 1.59 ± 0.96, respectively, *P* < 0.001).

**Table 1:** **Baseline characteristics of the study population (*n*** **= 708) according to urinary adiponectin tertile**

	**Tetile-1 (*n*** **= 236)**	**Tertile-2 (*n*** **= 236)**	**Tertile-3 (*n*** **= 236)**	***P*value**
	**(<3.19 μg/gCr)**	**(3.19-7.07 μg/gCr)**	**(≥7.07 μg/gCr)**	
Age (years)	53.69 ± 13.86*†	61.15 ± 11.65	63.02 ± 10.27	<0.001
Sex (Female/Male)	102/134	145/91	124/112	<0.001
Fasting plasma glucose (mmol/l)	7.03 ± 1.77†	7.47 ± 1.85‡	8.13 ± 2.48	<0.001
HbA1C (%)	6.51 ± 1.11†	6.75 ± 1.22‡	7.23 ± 1.54	<0.001
Total cholesterol (mmol/l)	4.88 ± 1.05	4.87 ± 0.89	4.95 ± 0.92	0.601
Triglycerides(mmol/l)	1.57 ± 1.04†	1.71 ± 1.02	1.82 ± 1.11	0.035
HDL-cholesterol (mmol/l)	1.31 ± 0.31	1.29 ± 0.31	1.33 ± 0.35	0.493
LDL-cholesterol (mmol/l)	2.82 ± 0.88	2.73 ± 0.77	2.76 ± 0.83	0.479
Fasting insulin (pmol/l)	60.73 ± 31.79	67.05 ± 32.25	66.99 ± 30.28	0.045
HOMA-IR	3.27 ± 1.97†	3.76 ± 2.28	4.11 ± 2.53	<0.001
hs CRP (mg/l)	0.22 ± 0.52	0.19 ± 0.43	0.23 ± 0.56	0.625
BMI (kg/m^2^)	24.76 ± 3.43	24.68 ± 3.40	24.93 ± 3.22	0.725
Waist circumference (cm)	85.28 ± 9.63	85.95 ± 8.77	87.11 ± 8.59	0.088
Systolic Blood Pressure (mmHg)	126.98 ± 15.04†	129.83 ± 15.36‡	135.12 ± 16.86	<0.001
Diastolic Blood Pressure (mmHg)	80.56 ± 10.78†	81.70 ± 10.39‡	84.87 ± 10.99	<0.001
eCcr (ml/min)	93.59 ± 26.32*†	86.67 ± 26.73	84.18 ± 27.32	0.001
Current alcohol intake (%)	22.0	15.7	21.6	0.156
Current smoker (%)	17.8	11.9	13.6	0.171
Taking ACEi/ARB medication (%)	14.8	19.9	19.9	0.249
Urinary A/C ratio (mg/g)	21.65 ± 34.80*†	30.48 ± 47.62	60.76 ± 64.85	<0.001
Serum adiponectin (μg/ml)	6.46 ± 5.06*†	8.36 ± 6.25	8.64 ± 6.61	0.001

All data are expressed as mean ± SD or percentages. *P* value = the difference among the three groups using ANOVA test. *P value <0.05 Tertile-1 vs. Tertile-2. †*P* value <0.05 Tertile-1 vs. Tertile-3. ‡*P* value <0.05 Tertile-2 vs. Tertile-3. (Post-hoc analysis using Bonferroni method).

*SD* standard deviation, *HbA1C* glycated hemoglobin, *HDL* high density lipoprotein, *LDL* low density lipoprotein, *HOMA-IR* Homeostasis model assessment of insulin resistance, *hs CRP* high sensitivity C-reactive protein, *BMI* body mass index, *eCcr* estimated creatinine clearance rate, *ACEi* ACE inhibitor, *ARB* Angiotensin receptor blocker, *A/C ratio* albumin/creatinine ratio.

### Relationship between urinary adiponectin level and various clinical parameters

Urinary adiponectin concentration was positively correlated with age, fasting plasma glucose level, HbA1C level, triglyceride level, HOMA-IR, systolic/diastolic blood pressure, mean baPWV, and urinary A/C ratio (all *P* < 0.05). After adjusting for age, sex, and eCcr, fasting plasma glucose level (r = 0.169, *P* < 0.001), HbA1C level (r = 0.167, *P* < 0.001), HDL-cholesterol level (r = 0.093, *P* = 0.023), systolic blood pressure (r = 0.112, *P* = 0.006), diastolic blood pressure (r = 0.121, *P* = 0.003), mean baPWV (r = 0.115, *P* = 0.005), and urinary A/C ratio (r = 0.352, *P* < 0.001) were found to be positively associated with urinary adiponectin concentration. Serum adiponectin concentration correlated with fasting plasma glucose level, HbA1C level, triglyceride level, HDL-C level, HOMA-IR, BMI, waist circumference, systolic blood pressure, and urinary A/C ratio (all *P* < 0.05) (Table 2).

**Table 2:** Correlations between adiponectin level and various clinical parameters

	*Urinary adiponectin(μg/gCr)		*Serum adiponectin(μg/ml)	
	**r**	***P*value**	**r**	***P*value**
Fasting plasma glucose	0.169	<0.001	−0.142	0.001
HbA1C	0.167	<0.001	−0.116	0.005
Total cholesterol	0.032	0.438	0.077	0.063
Triglycerides	0.058	0.160	−0.245	<0.001
HDL-cholesterol	0.093	0.023	0.283	<0.001
LDL-cholesterol	−0.012	0.769	0.062	0.135
HOMA-IR	0.078	0.057	−0.250	<0.001
BMI	−0.013	0.749	−0.121	0.003
Waist circumference	0.008	0.848	−0.113	0.006
Systolic blood pressure	0.112	0.006	−0.093	0.023
Diastolic blood pressure	0.121	0.003	−0.039	0.348
Mean baPWV	0.115	0.005	−0.036	0.383
Urinary A/C ratio	0.352	<0.001	−0.086	0.036
*Serum adiponectin	0.131	0.001	-	-
*Urinary adiponectin	-	-	0.131	0.001

Age-, sex-, and eCcr-adjusted model.

*Logarithmic transformation was performed due to a skewed distribution.

Mean baPWV value was calculated as (Right baPWV + Left baPWV)/2.

*HbA1C* glycated hemoglobin, *HDL* high density lipoprotein, *LDL* low density lipoprotein, *HOMA-IR* Homeostasis model assessment of insulin resistance, *BMI* body mass index, *baPWV* brachial-ankle pulse wave velocity, *A/C ratio* albumin/creatinine ratio, *eCcr* estimated creatinine clearance rate.

### Association between urinary adiponectin level and microalbuminuria

Multivariate logistic regression analysis was performed to calculate odds ratios (ORs) for the risk of microalbuminuria. We defined microalbuminuria as a urinary A/C ratio ≥ 30 mg/g. After adjusting for age, sex, and eCcr, the odds ratio for the risk of microalbuminuria in subjects in the highest tertile of urinary adiponectin concentrations was significantly greater than that of subjects in the lowest tertile (OR, 5.607; 95% CI, 3.562 to 8.828; *P* < 0.001). This result was significant after further adjusting for HbA1C level, triglyceride level, HDL-C level, BMI, blood pressure, HOMA-IR, alcohol/smoking habits, and ACE/ARB use, in addition to age, sex, and eCcr (OR, 5.142; 95% CI, 3.145 to 8.409; *P* < 0.001). This relationship remained significant even after adjusting for serum adiponectin level in addition to the other parameters (OR, 5.556; 95% CI, 3.334 to 9.258; *P* < 0.001) (Table 3). We also constructed receiver operating characteristics (ROC) curves based on urinary adiponectin concentration. The area under the ROC curve (AUC) of urinary adiponectin concentration for detecting microalbuminuria was significantly larger than that of serum adiponectin concentration (AUC of urinary adiponectin = 0.734; 95% CI, 0.693 to 0.776; AUC of serum adiponectin = 0.442; 95% CI, 0.396 to 0.488).

**Table 3:** Multivariable logistic regression analysis showing odds ratios for the risk of microalbuminuria according to urinary adiponectin tertile

	**Model 1**		**Model 2**		**Model 3**	
	**OR**	***P*value**	**OR**	***P*value**	**OR**	***P*value**
Tertile-1	1 (reference)		1 (reference)		1 (reference)	
Tertile-2	1.297 (0.800-2.103)	0.292	1.264 (0.758-2.109)	0.369	1.346 (0.796-2.274)	0.268
Tertile-3	5.607 (3.562-8.828)	<0.001	5.142 (3.145-8.409)	<0.001	5.556 (3.334-9.258)	<0.001

Model 1: adjusted for age, sex, eCcr.

Model 2: model 1 further adjusted for HbA1C, triglycerides, HDL-cholesterol, BMI, systolic blood pressure, diastolic blood pressure, HOMA-IR, current alcohol intake, current smoking, and ACEi/ARB use.

Model 3: model 2 further adjusted for serum adiponectin level.

*OR* odds ratio*, eCcr* estimated creatinine clearance rate, *HbA1C* glycated hemoglobin, *HDL* high density lipoprotein, *BMI* body mass index, *HOMA-IR* Homeostasis model assessment of insulin resistance, *ACEi* ACE inhibitor, *ARB* Angiotensin receptor blocker.

### Association between urinary adiponectin level and pulse wave velocity

Brachial-ankle pulse wave velocity (baPWV) data of 630 subjects were analyzed. PWV on both sides increased progressively with increased urinary adiponectin concentration. Mean baPWV increased significantly from the lowest to the highest urinary adiponectin tertile (*P* < 0.001) (Table 4). Univariate linear regression analysis showed that mean baPWV level is significantly associated with urinary A/C ratio (β = 1.286, *P* < 0.001) and urinary adiponectin concentration (β =2.642, *P* < 0.001), but not with serum adiponectin concentration (β =2.133, *P* = 0.317). Backward multiple linear regression analysis revealed that urinary adiponectin concentration was significantly associated with mean baPWV after controlling for confounding variables (R^2^ = 0.435, *P* = 0.043) (Table 5).

**Table 4:** **Pulse wave velocity of subjects categorized by urinary adiponectin concentration (*n*** **= 630)**

	**Urinary adiponectin (μg/gCr)**			***P*value**
	**Tertile-1**	**Tertile-2**	**Tertile-3**	
	**(<3.47)**	**(3.48-8.20)**	**(≥8.21)**	
Right baPWV (cm/sec)	1559.6	1594.1	1687.2	<0.001
	(1515.2-1603.9)	(1551.9-1636.3)	(1638.4-1736.0)	
Left baPWV (cm/sec)	1583.6	1605.1	1712.1	<0.001
	(1540.5-1626.7)	(1564.0-1646.1)	(1663.4-1760.8)	
Maximum baPWV (cm/sec)	1604.9	1632.7	1734.7	<0.001
	(1560.7-1649.1)	(1590.3-1675.1)	(1685.5-1784.0)	
Mean baPWV (cm/sec)	1570.6	1599.6	1698.8	<0.001
	(1527.5-1613.8)	(1558.4-1640.7)	(1650.6-1747.1)	

The mean baPWV value was calculated as (Right baPWV + Left baPWV)/2.

The maximum baPWV value was the higher of the two baPWV values.

Data are means (95% confidence intervals).

*baPWV* brachial-ankle pulse wave velocity.

**Table 5:** Linear regression analysis with mean baPWV as dependent variable

	**Univariate linear regression analysis**	
	**β (SE)**	***P*value**
Age(years)	16.616 (0.989)	<0.001
Triglyceride(mmol/l)	25.045 (12.563)	0.047
HOMA-IR	16.792 (5.806)	0.004
eCcr (ml/min)	−4.557 (0.442)	<0.001
Systolic blood pressure (mmHg)	8.955 (0.771)	<0.001
Urinary A/C ratio(mg/g)	1.286 (0.236)	<0.001
Serum adiponectin(μg/ml)	2.133 (2.132)	0.317
Urinary adiponectin (μg/gCr)	2.642 (0.671)	<0.001
	**Backward multiple linear regression analysis**	
	**β (SE)**	***P*value**
Age(years)	12.706 (1.303)	<0.001
Sex (Female/Male)	54.530 (21.360)	0.011
HOMA-IR	12.672 (4.585)	0.006
eCcr (ml/min)	−1.105 (0.513)	0.032
Systolic blood pressure (mmHg)	6.465 (0.680)	<0.001
Smoking status (yes)	56.481 (30.437)	0.064
Urinary adiponectin(μg/gCr)	1.085 (0.536)	0.043
R^2^	0.435	

Backward multiple linear regression analysis was used with mean baPWV as the dependent variable, and age, sex, HOMA-IR, triglyceride, eCcr, systolic blood pressure, urinary A/C ratio, smoking status, diabetes status, ACEi/ARB use, serum adiponectin, and urinary adiponectin as independent variables.

β is the unstandardized regression coefficient.

*SE* standard errors, *baPWV* brachial-ankle pulse wave velocity, *HOMA-IR* Homeostasis model assessment of insulin resistance, *eCcr* estimated creatinine clearance rate, *A/C ratio* albumin/creatinine ratio, *ACEi* ACE inhibitor, *ARB* Angiotensin receptor blocker.

## Discussion

We showed that increased urinary adiponectin concentration is significantly associated with microalbuminuria and increased mean baPWV. Our results indicate that urinary adiponectin may be a useful biomarker for detecting vascular damage such as microalbuminuria and arterial stiffness.

Several earlier studies focused on the relationship between plasma adiponectin and renal complications in diabetic patients [6,16]. Few researchers, however, have investigated the association between urinary adiponectin level and vascular damage. One study [11] assessed whether urinary adiponectin excretion might represent vascular damage in type 2 diabetic patients. One hundred fifty-six diabetic patients with a past history of diabetic nephropathy and 40 healthy subjects were evaluated in that study. The investigators found no significant relationship between urinary adiponectin and 24-hour urinary albumin excretion rate, but the urinary adiponectin level was significantly higher in diabetic patients than in healthy subjects. Furthermore, these researchers reported that adiponectinuria was associated with common carotid artery intima-media-thickness in the diabetic patients. In a study of diabetic patients, Koshimura et al. [12] showed that the urinary adiponectin excretion level was markedly higher in the group with overt nephropathy than in the group without nephropathy. However, these authors did not find a significant association with urinary adiponectin level in patients with normoalbuminuria and microalbuminuria, only among those with macroalbumi nuria (>300 mg/g). However, their study population comprised only 38 males with type 2 diabetes.

We found that urinary adiponectin concentration was significantly associated with the urinary A/C ratio and that there was a significant difference in urinary adiponectin concentration between subjects with normoalbuminuria and microalbuminuria. The correlation between urinary adiponectin and urinary A/C ratio was significant after adjusting for serum adiponectin level.

The molecular weight of an adiponectin monomer is approximately 28 kDa, while a dimer is 56 kDa and a trimer is 68 kDa [11]. Adiponectin circulates predominantly as a trimer (LMW), hexamer (MMW), or high molecular weight multimer (HMW). Shen et al. [17] demonstrated that urinary adiponectin is physiologically intact and mainly present as a trimer (LMW). The molecular weight of the trimeric form of adiponectin is similar to the molecular weight of albumin, which is about 67 kDa. Some reports have suggested that vascular complications precede the onset of albuminuria or the detection of diabetes [18,19]. Therefore, a measure of urinary total adiponectin in the monomeric to trimeric forms could be a useful tool for early detection of vascular changes due to its size, which is smaller than that of albumin. If it were possible to compare the results of renal biopsy specimens according to the grade of adiponectinuria or albuminuria, we could obtain more information on comparison between markers for the detection of diabetic nephropathy. Future studies will be required to clarify the pathologic evidence.

Furthermore, we provided evidence that measuring the urinary adiponectin level is more effective than measuring the serum adiponectin level in order to detect microalbuminuria. Measurement of urinary biomarkers is more useful than that of serum biomarkers, because urine can be sampled simply and non-invasively [14].

The mechanism through which plasma or urinary adiponectin excretion is related to diabetic nephropathy or other vascular complications is unclear. Adiponectin has been shown in previous studies to protect against vascular damage. Some reports, including an experimental study, revealed that adiponectin-deficient (*Ad*^*−/−*^) mice treated with adiponectin showed reduced albuminuria and improvement in podocyte function [20,21]. Sharma et al. [20] demonstrated that plasma adiponectin levels had a negative correlation with the degree of albuminuria in non-diabetic subjects. They suggested that plasma adiponectin is a regulator of albuminuria and modulates oxidant stress in podocyte foot processes. Shimotomai et al. [22] reported that increased urinary adiponectin level may be explained by altered glomerular permeability. Consistent with these hypotheses, we found that urinary adiponectin concentration positively correlated with the urinary A/C ratio, and that serum adiponectin concentration negatively correlated with the urinary A/C ratio. In our study, we observed that the mean serum adiponectin concentration increased with increasing urinary adiponectin tertiles. We could not determine the precise mechanism behind this observation. We observed that the mean urinary adiponectin concentration was higher in diabetic patients than in the normal glucose status group. Also, we found that the mean serum adiponectin concentration was lower in diabetic patients than in the normal glucose status group. If the mechanism were due to ‘spill-over’, then the serum adiponectin level would be similar to or higher in the diabetic group than in the normal glucose status group, in order to compensate for the renal loss of adiponectin. We speculate that it may be another disease pattern of the results; that is, serum adiponectin may not have compensated fully as urinary adiponectin level decreased.

We also found that increased urinary adiponectin level was associated with degree of arterial stiffness. Many researchers have assessed the association between plasma adiponectin level and arterial stiffness or blood pressure [7,23]. Some studies have found that decreased plasma adiponectin concentration was related to stiffness of both the central and peripheral arteries in hypertensive patients [8,23]. However, another study found no correlation between plasma adiponectin level and peripheral arterial stiffness [24]. In our study, we used brachial-ankle pulse wave velocity (baPWV) as a simple measure of arterial stiffness and as a predictive marker of macrovascular complications [25]. We found that urinary adiponectin concentration, but not serum adiponectin concentration, was an independent predictor of baPWV as a marker of arterial stiffness after adjusting for confounding factors such as age, lipid profile, and systolic blood pressure [26,27]. Recently, a few studies have appeared reporting that circulating adiponectin did not show a cardioprotective effect in animal models [28]. In humans, serum adiponectin concentrations rise across chronic heart failure stages, although this finding is less evident in type 2 diabetic patients according to the study by Baldasseroni et al. [29]. In our results, we did not find evidence of a protective role for serum adiponectin against vascular damage, in accord with these reports.

Our study has several strengths compared with previous urinary adiponectin studies. We performed our investigation in a heterogeneous population that included healthy normoglycemic subjects, prediabetic subjects, and type 2 diabetic patients. Additionally, we recruited a much larger study population than did previous studies. We excluded those subjects with macroalbuminuria and focused our assessment on the relationship between urinary adiponectin concentration and microalbuminuria.

Several limitations of our study must be considered when interpreting our results. First, our study was cross-sectional. Our findings are therefore limited to documenting causal relationship. Second, we measured total adiponectin concentration, and not that of individual isoforms. Some reports have suggested that high molecular weight adiponectin is a more powerful marker of insulin resistance and arterial stiffness than is low molecular weight adiponectin [8,30]. This may be the reason why we failed to find a significant relationship between serum adiponectin level and arterial stiffness in our study. Follow-up investigations are needed to examine the potential associations between adiponectin isoforms and vascular complications. Thirdly, we used baPWV as a representative tool for detecting arterial stiffness instead of aortic PWV, which is considered to be a better marker. Further studies are needed to validate the significance of baPWV in a larger prospective design.

## Conclusions

We demonstrated that increased urinary adiponectin concentration was related to an increased risk of microalbuminuria. Our results also revealed a significant association between urinary adiponectin concentration and vascular stiffness assessed using baPWV. These findings indicate that urinary adiponectin level can potentially be used as a noninvasive early biomarker of vascular dysfunction.

## Abbreviations

OR: Odds ratio; A/C ratio: Albumin/creatinine ratio; ACEi: ACE inhibitor; ARB: Angiotensin receptor blocker; baPWV: Brachial-ankle pulse wave velocity; eCcr: Estimated creatinine clearance rate; HOMA-IR: Homeostasis model assessment of insulin resistance; hs CRP: High sensitivity C-reactive protein; ROC: Receiver operating characteristic.

## Competing interests

JWP, NL, and B-SY are employees of AdipoGen, Inc. The other authors declare that they have no conflicts of interest associated with this manuscript.

## Authors’ contributions

WSJ collected and analyzed the data and wrote the manuscript. C-YP coordinated the study, interpreted the data, contributed to the discussion, and reviewed and edited the manuscript. JWP, NL, SEP, EJR, WYL, KWO, SWP, and B-SY contributed to the discussion and reviewed and edited the manuscript. C-YP and B-SY are guarantors of this work and, as such, had full access to all the data in the study and take responsibility for the integrity of the data and the accuracy of the data analysis. All authors read and approved the final manuscript.

## Authors’ information

Correspondence and reprint requests

All correspondence regarding the clinical aspects of this study should be forwarded to Professor Cheol-Young Park at the Division of Endocrinology and Metabolism, Department of Internal Medicine, Kangbuk Samsung Hospital, Sungkyunkwan University School of Medicine, Seoul, Republic of Korea, E-mail: cydoctor@chol.com. All correspondence regarding the technical aspects associated with this study should be submitted to Byung-Soo Youn, PhD, chief executive officer of AdipoGen, Rm 401, Venture Building B, Songdo Technopark, 7–50 Songdo-dong, Yeonsu-gu, Incheon, Republic of Korea, E-mail: bsyoun@adipogen.com.
